# Enhanced Interface with Strong Charge Delocalization toward Ultralow Overpotential CO_2_ Electroreduction

**DOI:** 10.1002/smsc.202300169

**Published:** 2023-11-27

**Authors:** Yu-Feng Tang, Tong Zhang, Hong-Cheng Mi, Mulin Yu, Peng-Fei Sui, Xian-Zhu Fu, Jing-Li Luo, Subiao Liu

**Affiliations:** ^1^ School of Minerals Processing and Bioengineering Central South University Changsha Hunan 410083 China; ^2^ Department of Chemical and Materials Engineering University of Alberta Edmonton Alberta T6G 1H9 Canada; ^3^ College of Materials Science and Engineering Shenzhen University Shenzhen Guangdong 518000 China

**Keywords:** charge delocalization, density functional theory, electrochemical CO_2_ reduction, enhanced interface effects, porous nanostructures

## Abstract

The construction of an interface has been demonstrated as one of the most insightful strategies for designing efficient catalysts toward electrochemical CO_2_ reduction (CO_2_RR). However, the weak interfacial interaction and inherent instability inevitably hinder a further performance enhancement in CO_2_RR attributable to the interface effect. Herein, 2 nm Ag nanoclusters (Ag NCs) are embedded onto CeO_2_ nanospheres (CeO_2_ NSs) with highly interconnected porosity (Ag NCs@CeO_2_ NSs) to exclusively study the pure interface effect toward CO_2_RR. The enhanced Ag–CeO_2_ pure interface endows Ag NCs@CeO_2_ NSs with a remarkably larger current density, significantly higher Faraday efficiency (FE), and energy efficiency as compared to Ag NCs, CeO_2_ NSs, and the one with Ag NCs dispersed on CeO_2_ nanoparticles. More importantly, an impressively high CO FE of over 70.0% is achieved at an ultralow overpotential (*η*) of 146 mV. The free energy and differential charge calculations, coupled with X‐ray photoelectron spectroscopy results jointly imply that the effective initiation of CO_2_RR to CO at a lower *η* over Ag NCs@CeO_2_ NSs derives from the enhanced interface‐induced charge delocalization, which enhances the electron transfer ability toward *COOH intermediate, thus overcoming the energy barrier demanded for the rate‐determining step.

## Graphical abstract

Herein, 2 nm Ag nanoclusters are embedded onto highly porous CeO_2_ nanospheres to exclusively study the pure interface effect toward CO_2_ reduction reaction. The enhanced Ag–CeO_2_ interface results in significant charge delocalization, which overcomes the energy barrier demanded for the rate‐determining step, leading to an impressive CO Faraday efficiency of over 70.0% at an ultralow overpotential of 146 mV.

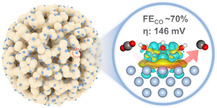

## 1 Introduction

Global climate change and the associated environmental issues are exacerbated by fossil fuel burning, which sharply increases CO_2_ emissions, the cause of global warming.^[^ ^1^ ^]^ To battle against the greenhouse effect, various technologies (e.g., thermochemistry,^[^ ^2^ ^]^ photochemistry,^[^ ^3^ ^]^ biochemistry,^[^ ^4^ ^]^ and electrochemistry^[^ ^5^ ^]^) have been employed with varying degrees of success to reduce CO_2_ emissions and simultaneously utilize CO_2_ as a carbon source to produce valuable carbon‐neutral chemicals/fuels. Among them, electrochemical CO_2_ reduction (CO_2_RR) into high‐energy‐density chemicals/fuels (e.g., CO, CH_4_, HCOOH, and C_2_H_4_), powered by renewable but intermittent energy sources (e.g., tide, solar, and wind), provides an attractive solution to both CO_2_ emissions control and energy‐demanding challenges toward a sustainable future for humankind.^[^ ^6^ ^]^ Despite the huge prospect, CO_2_RR usually suffers from thermodynamically high energy barriers, kinetically sluggish reaction rate, and undesirable selectivity toward target products, due to the high energy demand for transforming the extremely stable linear CO_2_ molecule to the bent radical anion, the complicated reaction pathways involving proton‐coupled electron transfer steps, and the competitive hydrogen evolution reaction (HER).^[^ ^7^ ^]^ Thus, it has never been more imperative to design and construct highly electroactive and stable nanomaterials capable of driving efficient CO_2_RR to high‐value target products.

To this end, substantial amounts of effort, such as size^[^ ^8^ ^]^ and morphology control,^[^ ^9^ ^]^ composition tuning,^[^ ^10^ ^]^ interface engineering,^[^ ^11^ ^]^ defect incorporation,^[^ ^12^ ^]^ and ligand modification,^[^ ^13^ ^]^ have been dedicated to devising high‐performance nanomaterials toward CO_2_RR. Among them, the effective construction of an interface, particularly between nanostructured metals and metal oxides, has been demonstrated to be one of the most intellectual strategies, since it not only preserves and stabilizes the active sites of nanostructured metals with excellent dispersions, but also introduces an interface effect. An extensive body of prior experiments (e.g., Au/CeO_2_,^[^ ^14^ ^]^ Bi/CeO_2_,^[^ ^15^ ^]^ In/In_2_O_3_,^[^ ^16^ ^]^ and Cu/In_2_O_3_^[^ ^17^ ^]^), coupled with persuasive theoretical computations, has revealed that atoms situated at interfacial sites are characterized by a coordination deficiency in comparison to their counterparts in the bulk phase, which are usually the active sites for the activation of CO_2_. Furthermore, in a diverse array of systems (e.g., Ag/SnO_*x*_,^[^ ^18^ ^]^ Ag/ZnO,^[^ ^19^ ^]^ Cu/ZrO_2_,^[^ ^20^ ^]^ and Cu/Ce(OH)_*x*_^[^ ^21^ ^]^), there has been a conspicuous observation of synergistic behavior at interfacial sites. This synergism, which is manifested through the regulation of charge distribution, facilitation of electron transfer and alteration in the binding energy to reaction intermediates consequently deliver an equivalent or even higher CO_2_RR performance with a much lower metal loading. Although it has been previously demonstrated with the aid of in situ characterization and theoretical calculations that the construction of metal–metal oxide interfaces can enhance CO_2_ adsorption and intermediates transformation on catalysts.^[^ ^14^, ^20^ ^]^ However, few reports have further explored the underlying mechanism of the role of interfacial sites for such an enhanced adsorption ability. What is even more critical is the difficulty in distinguishing pure interface effect from other influencing factors, which hinders an in‐depth mechanism understanding underpinning performance enhancement in CO_2_RR attributable to pure interface effect. To surmount the traditional bottlenecks of interface engineering, the prerequisites for creating pure metal–metal oxide interfacial sites capable of exclusively delving into the underlying mechanism of interface effect have normally depended on the compatibility and harmony between two components that constitute the interfacial sites, the plenitudinous exposure of active interfacial sites possessing favorable electronic structures, the elimination of interferences from other interfacial sites but the target ones, the strengthened stability of interfacial sites in a high‐energy states, and the sufficient porosity for mass transfer to expedite the kinetics.

Here, 2 nm Ag nanoclusters (Ag NCs, Figure S1, Supporting Information) were embedded onto CeO_2_ nanospheres (CeO_2_ NSs, Figure S2, Supporting Information) with highly interconnected porosity, denoted as Ag NCs@CeO_2_ NSs, to exclusively study the pure interface effect toward CO_2_RR. The results show that, due to the being‐tuned charge delocalization induced by the enhanced Ag–CeO_2_ pure interface, a significantly accelerated proton‐coupled electron transfer to form reaction intermediates and a reduced energy barrier to initiate rate‐determining step (RDS) was achieved, which enables Ag NCs@CeO_2_ NSs to be particularly active for CO formation, especially at an ultralow overpotential (*η*).

## 2 Results and Discussion

**Figure 1:**
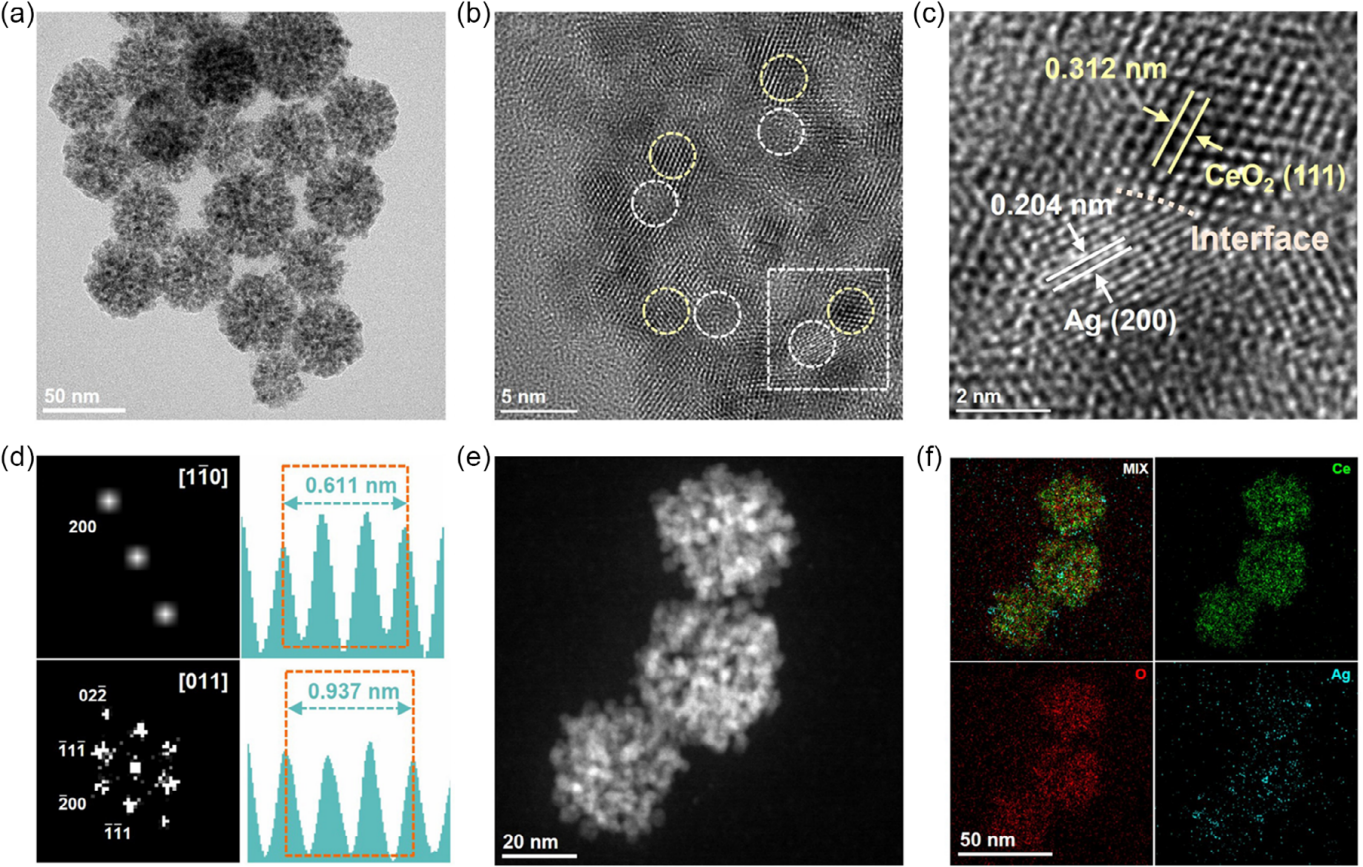
a) TEM and b) HRTEM images of Ag NCs@CeO_2_ NSs; c) enlarged square area in (b) showing the crystal lattices and d) the corresponding SAED patterns of individual Ag NC and ultrasmall CeO_2_ NP; e) HAADF‐STEM image and f) the associated EDX mapping profiles of Ag, Ce, and O on Ag NCs@CeO_2_ NSs.

The morphological structure of Ag NCs@CeO_2_ NSs was analyzed using bright‐field scanning transmission electron microscopy (STEM). Clearly, the CeO_2_ NSs with an average size of ≈50 nm are comprised of substantial ultrasmall CeO_2_ nanoparticles (NPs) with a crystallite size of ≈4 nm, which are inextricably interwoven with each other to form highly porous NSs shape (**Figure** **1**a,b). The specific NSs nanostructure undoubtedly could well preserve and stabilize the 2 nm Ag NCs with superb dispersions on the interconnected CeO_2_ NPs, thereby creating Ag–CeO_2_ pure interface and enhanced interfacial sites. A close inspection of the crystal lattice in the selected region (Figure 1b) illustrates that the two adjacent areas correspond to Ag NCs and CeO_2_ NSs with interplanar spacings of 0.204 and 0.312 nm (Figure 1c), respectively, which closely align with the values determined by X‐ray diffraction patterns (Figure S3, Supporting Information) for Ag(200) (PDF #04‐0783) and CeO_2_(111) (PDF #34‐0394). This persuasively demonstrates the successful construction of pure interface between Ag NCs and CeO_2_ NSs, as further confirmed by the corresponding selected area electron diffraction (SAED) pattern in Figure 1d. Concurrently, high‐angle annular dark field (HAADF)‐STEM, together with elemental mappings of energy‐dispersive X‐ray (EDX) spectroscopy, was also carried out, and the results validated that the uniformly distributed ≈2 nm species on CeO_2_ NSs were indeed Ag NCs (Figure 1e), as appeared in blue in Figure 1f. Apparently, the homogeneous dispersion guarantees an optimal utilization of Ag–CeO_2_ interfacial sites and presages an enhanced interface‐induced CO_2_RR.

**Figure 2:**
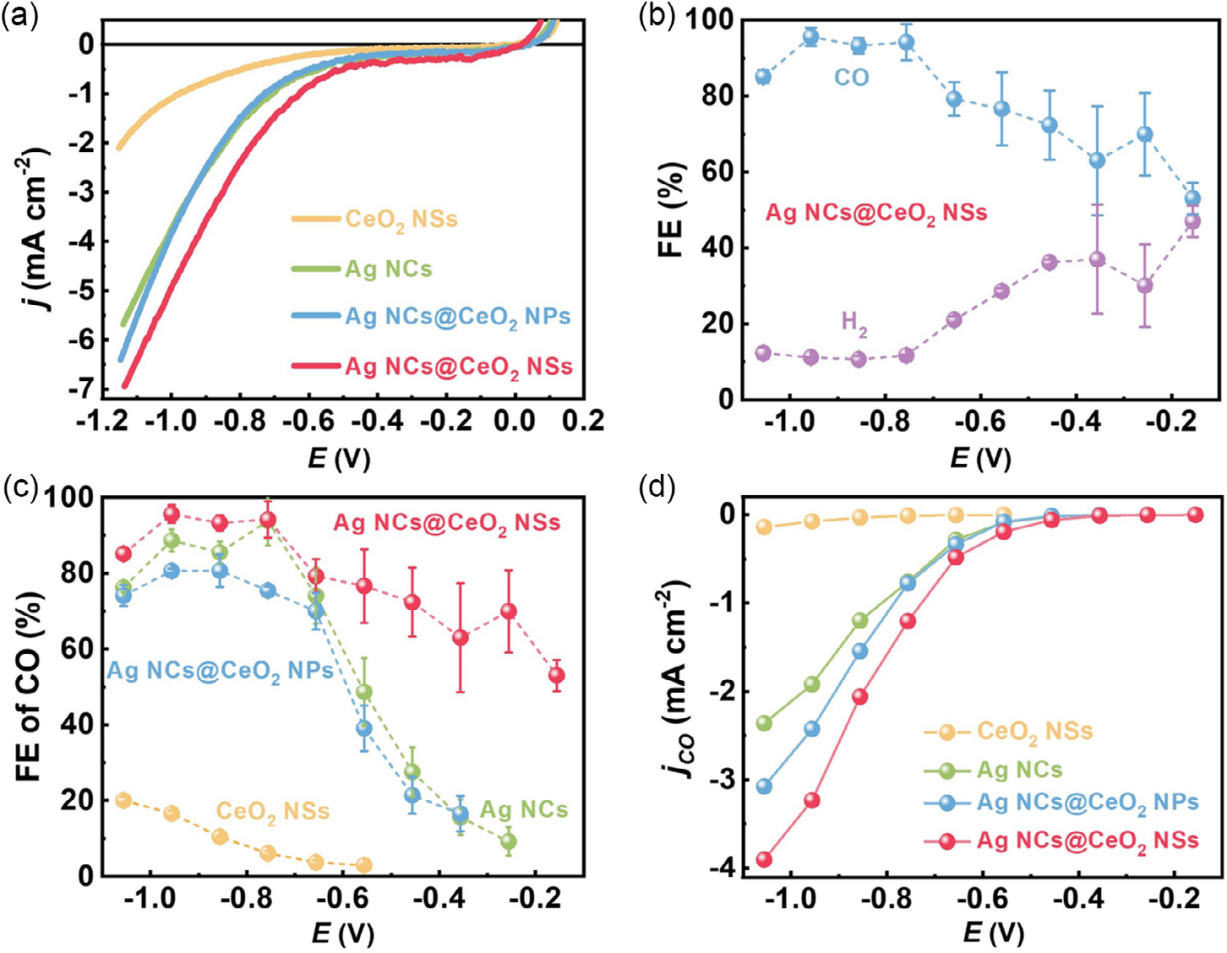
a) Cathodic LSV curves; b) FEs of all gaseous products on Ag NCs@CeO_2_ NSs; c) CO Fes; and d) *j*(CO) on Ag NCs@CeO_2_ NSs, Ag NCs, CeO_2_ NSs, and Ag NCs@CeO2 NPs at various potentials.

**Figure 3:**
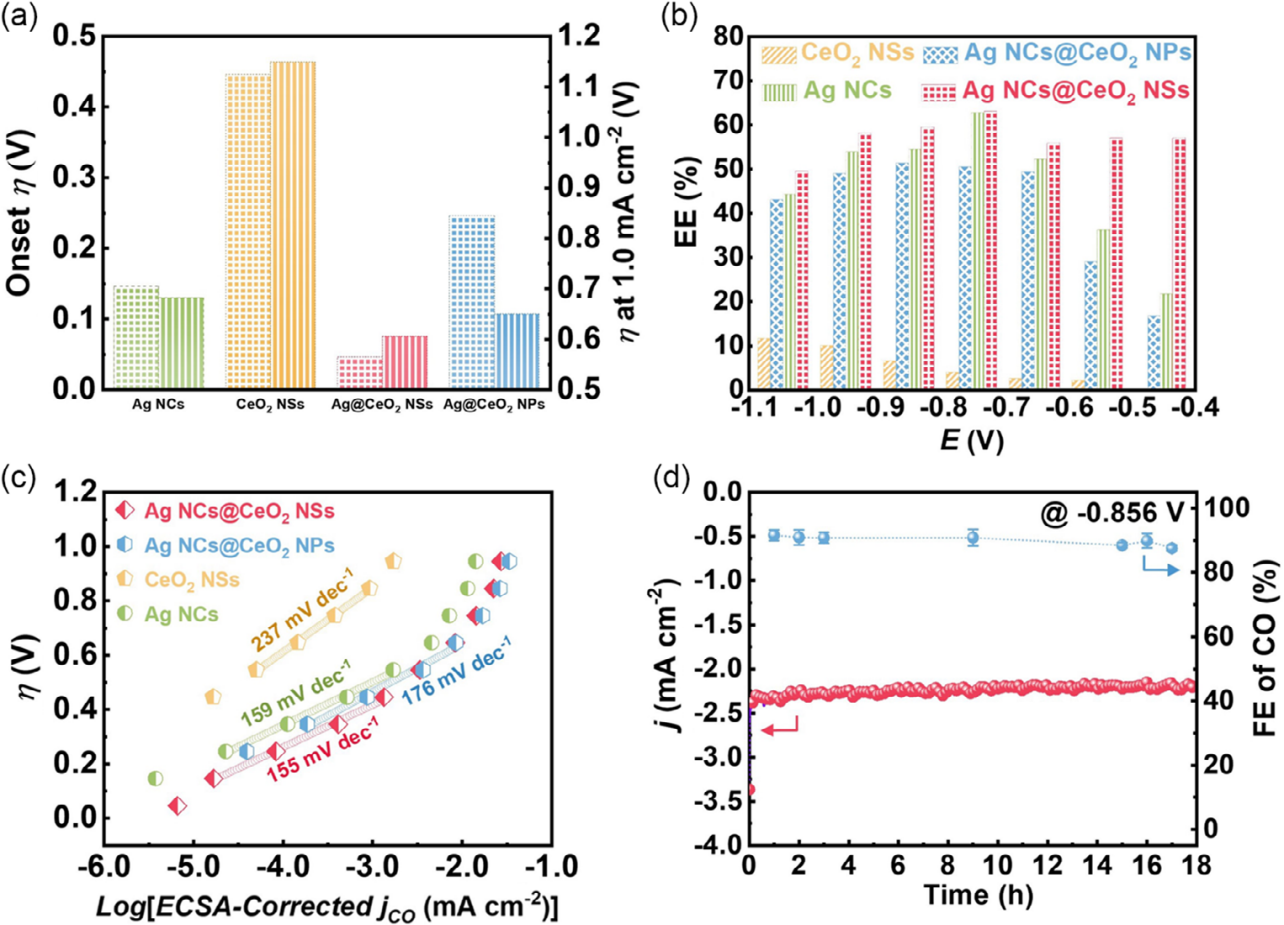
a) Onset *η* and *η* at 1.0 mA cm^−2^, b) EEs, and c) Tafel plots over Ag NCs@CeO_2_ NSs, Ag NCs, CeO_2_ NSs, and Ag NCs@CeO_2_ NPs; d) long‐term stability of Ag NCs@CeO2 NSs.

To evaluate CO_2_RR electrocatalytic activity for exclusively unraveling the enhanced Ag–CeO_2_ pure interface effect, Ag NCs@CeO_2_ NSs, coupled with Ag NCs, CeO_2_ NSs, and the one with Ag NCs dispersed on CeO_2_ NPs (Ag NCs@CeO_2_ NPs, Figure S4, Supporting Information), were all fabricated onto a glassy carbon electrode with a geometric area of 0.785 cm^2^ by an equivalent Ag loading. All the materials were examined in 0.1 m KHCO_3_ within a custom‐built cell separated by a Nafion‐117 membrane; all potentials are with reference to the reversible hydrogen electrode. Linear scanning voltammetry (LSV) measurements were first performed for all samples under Ar and CO_2_ atmospheres, respectively, to roughly investigate their electrocatalytic activity. Remarkably, the trend in current density (*j*) growth follows Ag NCs > Ag NCs@CeO_2_ NPs > Ag NCs@CeO_2_ NSs under Ar atmosphere as the applied potential increases (Figure S5, Supporting Information), whereas it exhibits a completely opposite trend under CO_2_ atmosphere (**Figure** **2**a), suggesting that the Ag–CeO_2_ interface is specifically active for CO_2_RR rather than HER. However, since the LSV results include current contributions from both CO_2_RR and the competitive HER, it only qualitatively concluded that CO_2_RR was favored on Ag NCs@CeO_2_ NSs. Thus, coupling CO_2_RR potentiostatic measurements at different potentials with product analyses for extracting partial *j*s toward target products is of prime importance to exclusively demonstrate the preferential CO_2_RR rather than HER over Ag NCs@CeO_2_ NSs. Gas chromatography and ion chromatography were employed to quantitatively determine the gaseous fuels and liquid products, respectively, and the results confirm that CO and H_2_ are the only two gaseous fuels (Figure 2b). A close observation of Faraday efficiencies (FEs) in Figure 2c finds that standalone CeO_2_ NSs possess an almost negligible ability to convert CO_2_ to CO with a maximum FE_CO_ of only 16.6% and a low *j* of ≈2.1 mA cm^-^^2^ at –1.156 V (Figure 2a **)**. However, the introduction of a modest quantity of Ag NCs on CeO_2_ NPs to engender Ag–CeO_2_ interface culminates in a considerably enhanced *j* of 6.4 mA cm^−2^, even surpassing that of 5.6 mA cm^−2^ on Ag NCs. Meanwhile, the maximum FE_CO_ significantly increases to 80.7%, preliminarily indicating that Ag–CeO_2_ interface could indeed bolster CO_2_RR electrocatalytic activity. Nevertheless, the highly porous Ag NCs@CeO_2_ NSs with interconnected channels result in additional increments in both *j*s and CO FEs as compared to Ag NCs@CeO_2_ NPs due to an enhanced Ag–CeO_2_ pure interface effect. Specifically, the Ag NCs@CeO_2_ NSs show higher FEs toward CO formation than all the counterparts at all potentials, and reach its maximum FE of 95.6% at –0.956 V, comparable with most of the state‐of‐the‐art Ag‐based and non‐noble–metal‐based nanomaterials.^[^ ^22^ ^]^ More importantly, Ag NCs@CeO_2_ NSs maintain CO FEs over 70% in an ultrawide potential range from –0.296 to –1.056 V, where, particularly, it reaches a FE_CO_ of ≈70.0% at an ultralow *η* of 146 mV, over 7‐fold higher than that of 9.2% on Ag NCs, superior to most of the previously reported catalysts (Figure S6, Supporting Information). The significantly enhanced Ag–CeO_2_ pure interface effect is further verified by CO partial *j*s [*j*s_(CO)_], which was determined by integrating *j*s obtained at various potentials (Figure S7, Supporting Information) and CO FEs (Figure 2c). Clearly, a *j*_(CO)_ of over 3.90 mA cm^−2^ on Ag NCs@CeO_2_ NSs was obtained at –1.056 V, almost 1.3‐ and 1.6‐fold larger than that of 3.07 mA cm^−2^ on Ag NCs@CeO_2_ NPs and that of 2.36 mA cm^−2^ on Ag NCs (Figure 2d), respectively. In addition, a remarkably ultralow onset *η* of 46 mV and a *η* of 0.60 V to deliver a *j*_(CO)_ of 1.0 mA cm^−2^ (**Figure** **3**a) was achieved over Ag NCs@CeO_2_ NSs, comparably lower than those over Ag NCs, CeO_2_ NSs, and Ag NCs@CeO_2_ NPs. Furthermore, the low *η* together with the high FE of Ag NCs@CeO_2_ NSs contributes to maximum energy efficiency (EE) of 63.1% at –0.756 V (Figure 3b), and particularly achieves a comparably higher EE of 57.0% at an ultralow potential of –0.456 V, much higher than those over Ag NCs (21.7%) and Ag NCs@CeO_2_ NPs (16.8%), which is also comparable with most of the benchmarking nanomaterials for CO formation,^[^ ^23^ ^]^ further emphasizing the positively enhanced pure interface effect toward CO_2_RR.

To exclude the influences caused by different morphologies and sizes, and consequently confirm the intrinsic origins credited for the remarkably enhanced electrocatalytic activity of Ag NCs@CeO_2_ NSs for CO_2_RR, electrochemical surface areas (ECSAs) of Ag NCs@CeO_2_ NSs, Ag NCs, CeO_2_ NSs, and Ag NCs@CeO_2_ NPs were evaluated, and the results in Figure S8 and S9, Supporting Information show that Ag NCs@CeO_2_ NSs obtained a double layer capacitance (*C*_dl_) of 2.88 mF cm^−2^, approximately 0.86 that on Ag NCs (3.34 mF cm^−2^). However, the obtained *j*_(CO)_ at –1.056 V over Ag NCs@CeO_2_ NSs was 2.36 times higher than Ag NCs, delivering an intrinsically 2.74‐fold higher electrocatalytic activity. This, beyond any doubt, points out that the intrinsic activity of Ag NCs@CeO_2_ NSs originates from the massive highly active Ag–CeO_2_ interfacial sites. To further clarify the reaction kinetics, an ECSA‐normalized Tafel plot was thus illustrated in Figure 3c. As compared to the Tafel slopes of 159 mV dec^−1^ on Ag NCs, 237 mV dec^−1^ on CeO_2_ NSs, and 176 mV dec^−1^ on Ag NCs@CeO_2_ NPs, a much lower Tafel slope of 155 mV dec^−1^ was achieved over Ag NCs@CeO_2_ NSs, much closer to the theoretical value of 120 mV dec^−1^, indicative of higher intrinsic electrocatalytic activity of Ag NCs@CeO_2_ NSs toward the key ${\text{CO}}_{2}^{•¯}$ intermediate formation over the special architecture.^[^ ^24^ ^]^ This was further verified by electrochemical impedance spectroscopy results in Figure S10, Supporting Information, where Ag NCs@CeO_2_ NSs exhibited a smaller interface charge transfer resistance, indicating that Ag–CeO_2_ interface facilitates charge transfer to reaction intermediates more effectively during CO_2_RR. The long‐term stability, as a crucial parameter to evaluate practicability, was further assessed by recording the *j* as a function of time at a constant potential of –0.856 V. Remarkably, Ag NCs@CeO_2_ NSs displayed a slight CO FE decay of below 4.4% during the stability test (Figure 3d), significantly lower than Ag NCs@CeO_2_ NPs (16.1%) and Ag NCs (10.3%) (Figure S11, Supporting Information). The superior stability of Ag NCs@CeO_2_ NSs in terms of *j* and CO FE can be attributed to the anchoring effect of CeO_2_ NSs with abundant mesoporous channels on Ag NCs, which, to some extent, enhances the stability of high‐energy Ag–CeO_2_ interfacial sites.

**Figure 4:**
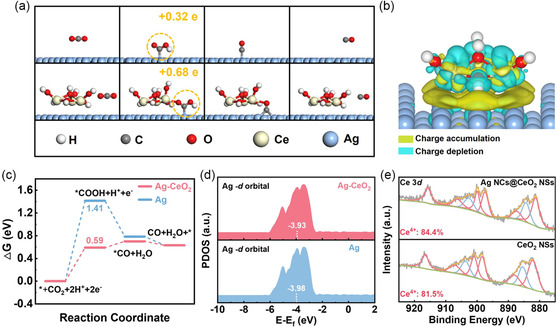
a) Optimized structures for key intermediates on Ag(100) and Ce_3_O_7_H_7_‐Ag(100) with labeled Bader charges; b) calculated differential charge density of Ce_3_O_7_H_7_‐Ag(100); c) free‐energy diagrams over Ag(100) and Ce_3_O_7_H_7_‐Ag(100) for CO_2_RR to CO; d) PDOS of *d*‐band for Ag in Ag(100) and Ce_3_O_7_H_7_‐Ag(100); e) Ce 3*d* spectra of Ag NCs@CeO_2_ NSs and CeO_2_ NSs.

To theoretically deepen the enhanced pure interface effect over Ag NCs@CeO_2_ NSs for CO_2_RR, density functional theory (DFT) calculations were conducted using a structure of Ce_3_O_7_H_7_‐Ag(100) (Figure S12, Supporting Information) to simulate Ag–CeO_2_ pure interface,^[^ ^14^ ^]^ and the computational hydrogen electrode model was employed to calculate the free energy diagrams. As depicted in **Figure** **4**a, the pathways through which CO_2_ is transformed to CO via a carboxyl intermediate (*COOH) coupled with two protons and electrons were analyzed on Ce_3_O_7_H_7_‐Ag(100) and Ag(100). Evidently, the formation of the key intermediate of *COOH, involving the first proton‐coupled electron transfer (i.e., CO_2_ + H^+^ + e^-^ → *COOH), encounters a comparably high‐energy barrier of 1.41 eV on Ag(100), which inevitably accumulates in the initial *η* for CO formation as the RDS. In contrast, the Δ *G* required to form the key *COOH intermediate on Ce_3_O_7_H_7_‐Ag(100) is only 0.59 eV, approximately 41.8% of that on Ag(100), indicating that the enhanced Ag–CeO_2_ pure interface can initiate CO_2_RR at lower applied potentials, which is consistent with previously observed high CO FEs achieved at ultralow *η* (Figure 4c). The interactions and electronic structure between Ce_3_O_7_H_7_ cluster and Ag(100) facet were also investigated by calculating differential charge density maps. A conspicuous decrease in charge density was observed on both Ce and Ag atoms, accompanied by a corresponding accumulation of charge throughout the entire interface (Figure 4b), suggesting that the construction of Ce_3_O_7_H_7_‐Ag(100) pure interface induces a strong charge delocalization from Ag and Ce atoms toward the interface region, which is believed to be the origin credited for the high electrocatalytic activity at the interfacial sites.^[^ ^19^ ^]^ As a result, the significantly increased charge density at the interfacial sites dramatically boosts the electronic supply ability to the reaction intermediates. Moreover, the Bader charge analyses provide quantitative evidence that Ce_3_O_7_H_7_‐Ag(100) transfers 0.68 electron to the *COOH intermediate, more than twice the 0.32 electron delivered by Ag(100) (Figure 4a), which is further supported by the projected density of states (PDOS) calculations. In comparison to the Ag(100), although the Ag in Ce_3_O_7_H_7_‐Ag(100) remains in the metallic state, its *d*‐band center shifts positively toward the Fermi level (Figure 4d), resulting in a stronger binding ability to the *COOH intermediate, and hence benefiting the CO production. Likewise, X‐ray photoelectron spectroscopy (XPS) results also confirm the existence of the metallic state of Ag in Ag NCs@CeO_2_ NSs (Figure S13, Supporting Information), indicating that the construction of Ag–CeO_2_ interface did not change the metallic nature of Ag. More importantly, the spectra of Ce 3*d* XPS show that the presence of Ce in Ag NCs@CeO_2_ NSs, Ag NCs@CeO_2_ NPs, CeO_2_ NSs, and CeO_2_ NPs is in the form of a mixture of Ce^4+^ and Ce^3+^, where the atomic fraction of Ce^4+^ increases from ≈81.5% in CeO_2_ NSs to ≈84.4% in Ag NCs@CeO_2_ NSs (Figure 4e). Apparently, the increase in Ce^4+^ concentration is not caused by the oxidizing agents during synthesis but induced by the enhanced strong interfacial interaction between Ag and CeO_2_, which facilitates the delocalization of electrons initially bound within Ce atoms to the interfacial sites, leading to an elevation of the valence. Therefore, it is concluded that the impressive CO_2_RR to CO at ultralow *η* over Ag NCs@CeO_2_ NSs stems from the enhanced interface‐induced charge delocalization, which significantly accelerates electron transfer to the key *COOH intermediate, and consequently mitigates the energy barrier required for the RDS.

## 3 Conclusion

In summary, Ag NCs@CeO_2_ NSs with enhanced Ag–CeO_2_ pure interface were successfully synthesized and found that Ag NCs@CeO_2_ NSs could effectively promote CO_2_RR to CO with remarkably higher electrocatalytic activity, near‐unity selectivity, and superior long‐term stability relative to Ag NCs, CeO_2_ NSs, and Ag NCs@CeO_2_ NPs. More importantly, Ag NCs@CeO_2_ NSs could initiate CO_2_RR at an impressively low *η*, together with a considerably high FE_CO_ in 0.1 m KHCO_3_. DFT calculations, coupled with differential charge calculation on the interface model, Bader analyses on key intermediate adsorption models, and XPS results, collectively suggest that the effective activation of CO_2_RR to CO at a lower *η* over Ag NCs@CeO_2_ NSs originates from the enhanced interface‐induced charge delocalization. This phenomenon significantly increases the electron density at interfacial sites and considerably enhances the electron transfer ability toward the key *COOH intermediate, thus strongly reducing the energy barrier demanded for the RDS. This study, thus, paves an avenue to construct enhanced pure interface through intellectually customizing specific metal oxide nanostructures capable of well preserving, dispersing, and stabilizing metal nanostructures.

## Conflict of Interest

The authors declare no conflict of interest.

## Supporting information

Supplementary Material

## Data Availability

The data that support the findings of this study are available from the corresponding author upon reasonable request.
