# Supplementary material for: Enhanced Interface with Strong Charge Delocalization toward Ultralow Overpotential CO2 Electroreduction
Source: Small Sci. 2023 Nov 27;4(1):2300169. doi: 10.1002/smsc.202300169 (PMC11935060; doi:10.1002/smsc.202300169)
Supplement: Supplementary file 1 — Supplementary Material [file SMSC-4-2300169-s001.pdf]

Supporting Information

Enhanced Interface with Strong Charge Delocalization toward Ultralow Overpotential CO_2 Electroreduction

*Yu-Feng Tang, Tong Zhang, Hongcheng Mi, Mulin Yu, Peng-Fei Sui, Xian-Zhu Fu, Jing-Li Luo, Subiao Liu**

Specifications of Chemicals and Gases

Silver (I) acetate (99%), cerium nitrate hexahydrate (99%), oleic acid (99%), 1-octadecene (99%), oleylamine (80%~90%), ethylene glycol (99%), propionic acid (99%), hexane (98.5%), acetic acid (99%), ethanol (99%), potassium bicarbonate (99%) and Nafion perfluorinated ion-exchange resin solution (5 wt% in mixture of lower aliphatic alcohol & H_2O) were purchased from Sigma-Aldrich. Nafion® N-117 membrane (0.18 mm thick) was purchased from Alfa Aesar; The glassy carbon electrode (GCE, 0.785 cm^2) was purchased from AIDA Science Technology Company, China. Deionized water was taken from a Millipore Autopure system. All chemicals are of analytical grade and used without further purification. Hydrogen (H_2, 99.999%), argon (Ar, 99.999%), compressed air (extra dry) and carbon dioxide (CO_2, 99.999%) were purchased from Air Liquide.

Experimental Details

Synthesis of silver nanoclusters (Ag NCs)

In a typical experiment, 0.168 g of silver (I) acetate was magnetically stirred in 1-octadecene (10 mL), oleic acid (4.5 mL), and oleylamine (0.5 mL) under a gentle N_2 flow. The mixture was first heated to 60 °C to form a homogeneous solution, and then gradually heated to 180 °C with a ramping rate of 3~5 °C min^{-1}. During keeping the temperature of 180 °C for 20 min, the solution gradually turned from transparent color into brown and consequently dark-brown color. The Ag NCs were separated by adding isopropanol (30 mL) when the solution was cooled down to room temperature, followed by a centrifugation step with a rotating rate of 10,000 rpm for 10 min. The obtained Ag NCs were finally dispersed in hexane for further use.

Synthesis of CeO_2 nanospheres (CeO_2 NSs) and CeO_2 nanoparticles (CeO_2 NPs)

The CeO_2 NSs were synthesized via a one-pot hydrothermal method. Specifically, 1.0 g cerium nitrate hexahydrate were dissolved in 1 mL deionized water, followed by the incorporation of 1 mL of propionic acid and 30 mL of ethylene glycol while stirring continuously to ensure a homogenous solution. Once finished, the solution was sealed in an autoclave and heated to 180 °C for 200 minutes before the resultant products were isolated through centrifugation at 10,000 rpm for 10 minutes, then washed with water and ethanol to remove residual ions and organic solvents, and finally air-dried at 80 °C to yield the finished products. The CeO_2 NPs were prepared by the thermal decomposition of cerium nitrate hexahydrate at 550 °C under air for 4 hours.

Synthesis of Ag NCs@CeO_2 NSs and Ag NCs@CeO_2 NPs

Ag NCs@CeO_2 NSs and Ag NCs@CeO_2 NPs were prepared through an ultrasonic loading method. Specifically, 75 mg of CeO_2 NSs were suspended in 10 mL of hexane and sonicated for 15 min. Then around 25 mg of Ag NCs in hexane were taken and introduced dropwise into the CeO_2 NSs-containing hexane solution under sonication. Once finished, the mixed suspension was continuously sonicated for 1 h to guarantee a complete Ag NCs adsorption by the CeO_2 NSs. The mixture was centrifuged (5000 rpm for 6 minutes) and washed with ethanol for three times, and the final products were dried in a vacuum furnace. To obtain Ag NCs@CeO_2 NPs, replace CeO_2 NSs with CeO_2 NPs while keeping other conditions unchanged.

Characterizations of materials

Microstructures were determined with a high-resolution Zeiss Sigma field emission scanning electron microscope (FESEM) equipped with an EDX detector and an EBSD detector. The low- and high-resolution transmission electron microscopy (TEM) images of both samples were acquired by using a Thermo Scientific Talos F200X STEM. The TEM uses an electron beam as light source (Lab6 crystal in this case) and the beam goes through (transmission) the specimen. The TEM was operated at an accelerating voltage of 200/300 kV. Images were achieved using a Gatan digital imaging system through Digital Micrograph software. X-ray photoelectron spectroscopy (XPS, Kratos AXIS Ultra) was used to investigate the surface chemistry of Ag NCs@CeO_2 NSs, Ag NCs, CeO_2 NSs, Ag NCs@CeO_2 NPs and CeO_2 NPs with the adventitious carbon (C 1s) at the binding energy (BE) of 284.6 eV as the reference.

Electrochemical measurements

Certain amounts of catalyst and acetylene black (the addition amount of Ag is 2.5 mg) were dispersed in 1 mL mixed solvent containing 700 μL isopropanol, 100 μL of 5 wt% Nafion solution and 200 μL deionized water to form a homogeneous ink after at least 3 h ultrasonic. Then the catalyst ink was loaded onto the GCE (0.785 cm^2), the GCE were mechanically polished with alumina paste before loading the catalyst suspension, then the GCE was dried overnight in air at room temperature before testing. The LSV curves were conducted with a Solartron 1255 frequency response analyzer and a Solartron 1286 electrochemical interface instrument in a home-made electrochemical cell using saturated calomel electrode as the reference electrode, a platinum gauze as the counter electrode and the catalyst coated GCE as the working electrode. The catalyst coated GCE was used as the working electrode, together with saturated calomel electrode as the reference electrode and platinum gauze as the counter electrode. The 0.1 M $KHCO_3$ electrolyte was saturated by bubbling CO_2 prior to the start of each experiment at a flow rate of 20 mL min^{-1} for 30 min until a saturated CO_2 condition was reached to ensure that all the oxygen was removed from the electrolyte. The flow of CO_2 was continuously maintained during the recording of LSV curves. The LSV test was repeated at least 20 times before data were recorded at a scan rate of 20 mV s^{-1}. All potentials are reported with respect to the reversible hydrogen electrode (RHE).

$$\text{Potential (V vs. RHE)} = \text{Potential (V vs. SCE)} + 0.241\ \text{V} + 0.0592 \times \text{pH}$$

Products analysis

The catalyst ink was loaded onto the GCE (0.785 cm^2) with a loading of ~0.637 mg cm^{-2} and a fresh catalyst was employed for each potential. The gas products from the cathode compartment were analyzed using a Hewlett-Packard model Agilent 7890B gas chromatograph (GC) equipped with a packed bed column (HaySep D) operated at 80 °C with a thermal conductivity detector and a flame ionization detector. Argon (Air liquid 5.0) was employed as carrier gas with a flow rate of 30 mL min^{-1}. The gaseous products, i.e., H_2 and CO were separated in a molecular sieve column (Alltech, part no. 57732, 1.65 m × 1/8 inch, molecular sieve 13X, 60/80 mesh) and hydrocarbons and CO_2 in a HaySep column (Alltech, part no. 14487, 3.5 m × 1/8 inch, HaySep D, 80/100 mesh). IC was used to quantify the concentration of liquid-phase products.

Calculation of Faraday efficiency (FE)

$$FE_j = \frac{2FVvp_0}{RT_0 I_{total}} \times 100\%$$

$$\mathrm{FE_j} = \frac{2\times 96{,}485\ \left(\frac{\mathrm{C}}{\mathrm{mol}}\right)\times \mathrm{V}\left(\frac{\mathrm{m^3}}{\mathrm{s}}\right)\times \mathrm{v(vol\%)}\times 1.01\times 10^5\left(\frac{\mathrm{N}}{\mathrm{m^2}}\right)}{8.314\left(\frac{\mathrm{N\ m}}{\mathrm{mol\ K}}\right)\times 298.15(\mathrm{K})\times \mathrm{I_{total}}\left(\frac{\mathrm{C}}{\mathrm{s}}\right)}\times 100\%$$

$$\mathrm{FE_j} = \frac{2\times 96{,}485\ \left(\frac{\mathrm{C}}{\mathrm{mol}}\right)\times \mathrm{V}\left(\frac{\mathrm{mL}}{\mathrm{min}}\right)\times 10^{-6}\left(\frac{\mathrm{m^3}}{\mathrm{mL}}\right)\times \mathrm{v(vol\%)}\times 1.01\times 10^5\left(\frac{\mathrm{N}}{\mathrm{m^2}}\right)}{8.314\left(\frac{\mathrm{N\ m}}{\mathrm{mol\ K}}\right)\times 298.15(\mathrm{K})\times \mathrm{I_{total}}\left(\frac{\mathrm{C}}{\mathrm{s}}\right)\times 60\left(\frac{\mathrm{s}}{\mathrm{min}}\right)}\times 100\%$$

$$\mathrm{FE_j} = \frac{0.1315\times \mathrm{V}\left(\frac{\mathrm{mL}}{\mathrm{min}}\right)\times \mathrm{v(vol\%)}}{\mathrm{I_{total}(A)}}\times 100\%$$

Where,

v (vol%) is the volume concentration of CO/H_2 in the outlet gas from the gas-tight cell,

V (mL min^{-1}) is the gas flow rate measured by a flowmeter at the exit of the cell at room temperature,

I_{total} (A) is steady-state cell current.

Electrochemical surface area (ECSA) measurements

ECSA $= R_f$S, where S stands for the real surface area of the smooth metal electrode, which is generally equal to the geometric area of GCE. The roughness factor R_f was estimated from the ratio of double-layer capacitance Cd_l for the working electrode and the corresponding smooth metal electrode (assuming that the average double-layer capacitance of a smooth metal surface is 20 uF cm^{-2}), that is, R_f=Cd_l/20 µF cm^{-2}. The Cd_l was determined by measuring the capacitive current associated with double-layer charging from the scan rate dependence of cyclic voltammetric stripping. The potential window of cyclic voltammetric stripping was –0.3 V to –0.2 V versus SCE (0.1 M $KHCO_3$). The was estimated by plotting the Δj=(j_a−j_c) at –0.25 V (where j_a and j_c are the anodic and cathodic current densities, respectively) versus SCE against the scan rate (i.e., 20, 40, 60, 80 and 100 mV s^{-1}).

Calculation of energy efficiency (EE)

$$\mathrm{EE_j} = \frac{\mathrm{E_j^0}\times \mathrm{FE_j}}{\mathrm{E_j^0}+\eta}\times 100\%$$

Where,

E_j^0 (V) is the equilibrium cell potential for a certain product,

FE_j (%) is the Faraday efficiency of the aiming product j,

η (V) is the overpotential.

Density function theory (DFT) calculations

Spin polarized DFT calculations were performed with the Perdew-Burke-Ernzerhof (PBE) exchange-correlation functional[1] in a plane wave pseudopotential implementation[2] using the Vienna ab initio simulation packages (VASP).[3] A cutoff energy of 400 eV for the plane-wave basis set was employed. The method of Methfessel-Paxton (MP) with a smearing width of 0.20 eV was adopted for transition metal surfaces and interfaces.[4] The DFT+U formalism was used to describe the localized (strongly correlated) 4f electrons in cerium.[5] For Ce, $U_{Ce} - J_{Ce} = 4.5$ eV was used, which has been successfully used in previous works to describe supported Ce-based species on transition metal surfaces.[6] Ag-CeO_2 was described by an inverse model system, a $Ce_3O_7H_7$ cluster deposited on a four-layer Ag(100) slab with 6×6 arrangement on each layer.[7] A (2×2×1) Gamma centered k-point sampling was adopted with a 15 Å vacuum to separate the slab perpendicular to the surface, where the bottom two layers were fixed as truncated bulk structure during structure optimization. The upper most layer of Ag (100) slab and the $Ce_3O_7H_7$ were fully relaxed with the adsorbates. For comparison, a (6×6) Ag (100) four-layer slab with a (2×2×1) k-points sampling was used. The convergence criteria for the residual force and energy during structure relaxation were set to 0.01 eV/Å and 1×10^{-5} eV, respectively. Following the previous studies,[8] CO_2RR was described by

$$CO_2 + * + H^+ + e^- \rightarrow *COOH$$

$$*COOH + H^+ + e^- \rightarrow *CO + H_2O$$

$$*CO \rightarrow * + CO$$

Free energy for each intermediate state was calculated via $\Delta G = \Delta E + \Delta ZPE - T\Delta S$, where ΔE is the reaction energy change based on the total energies from DFT calculations, ΔZPE is the variation in zero-point energies (ZPE) and ΔS is the change in entropy before and after the reaction. T is temperature and equals to 298 K. Following the previous studies for CO_2RR to CO, the proton-electron pair was assumed and the corresponding free energy was expressed using the reversible hydrogen electrode.[9]

To better understand the interaction between $Ce_3O_7H_7$ cluster and Ag(100), the differential charge density $\Delta\rho(r)$ was calculated via $\Delta\rho(r) = \Delta\rho_{Ce\text{-}Ag}(r) - \Delta\rho_{Ce}(r) - \Delta\rho_{Ag}(r)$, where $\Delta\rho_{Ce\text{-}Ag}(r)$, $\Delta\rho_{Ce}(r)$ and $\Delta\rho_{Ag}(r)$ are the electron density of $Ce_3O_7H_7$-Ag(100), $Ce_3O_7H_7$ cluster and Ag(100), respectively. Additionally, the Bader charge analyses was conducted to quantitatively determine the electron gain and loss information of * COOH intermediates on $Ce_3O_7H_7$-Ag(100) and Ag(100).

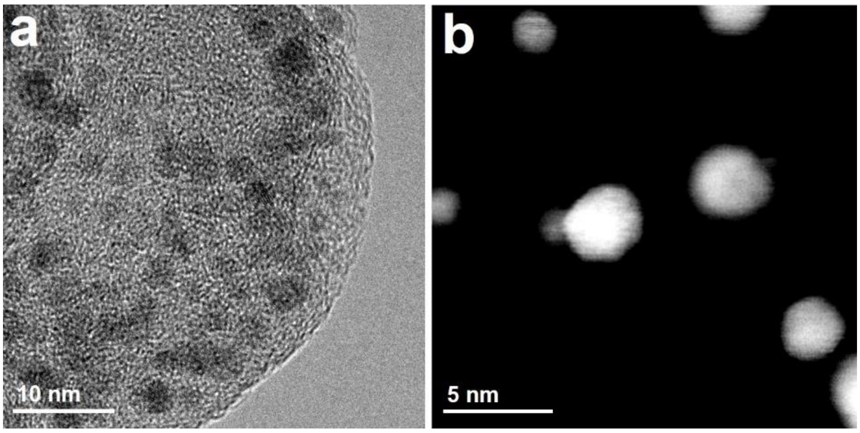

Figure S1. (a) HRTEM and (b) HAADF-STEM images of Ag NCs.

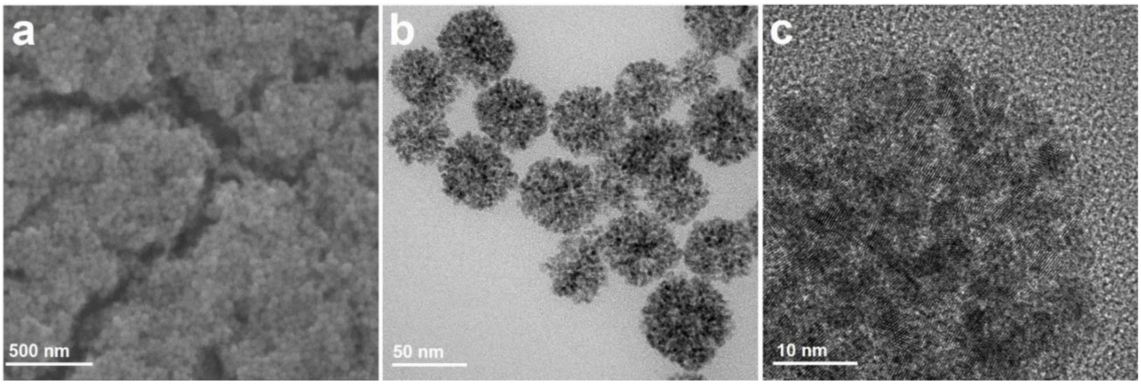

Figure S2. (a) FESEM and (b, c) TEM images of CeO_2 NSs.

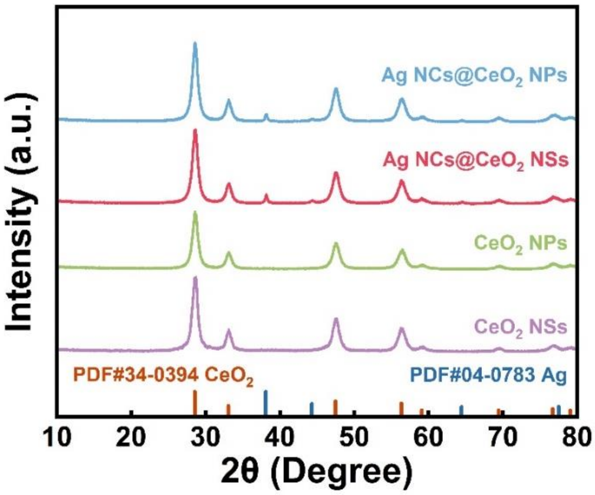

Figure S3. XRD results of CeO_2 NSs, CeO_2 NPs, Ag NCs@CeO_2 NSs and Ag NCs@CeO_2 NPs.

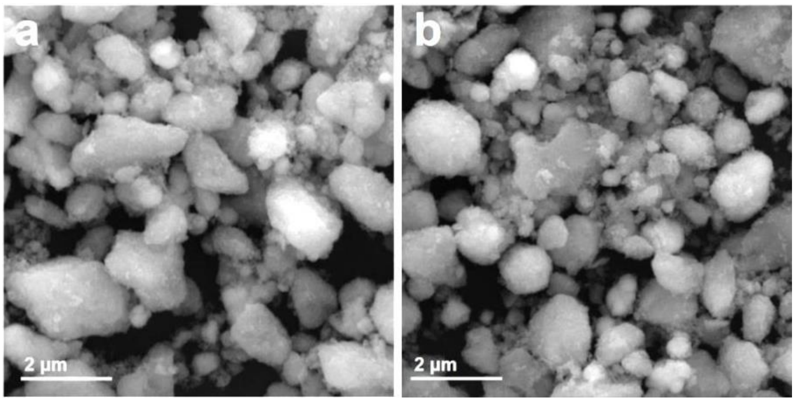

Figure S4. FESEM images of (a) CeO_2 NPSs and (b) Ag NCs@CeO_2 NPs.

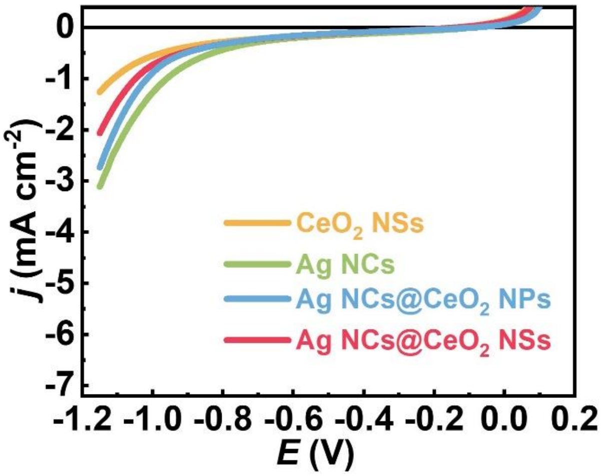

Figure S5. Cathodic LSV curves on Ag NCs@CeO_2 NSs, Ag NCs@CeO_2 NPs, Ag NCs, and CeO_2 NSs in Ar-saturated 0.1 M $KHCO_3$ aqueous solution.

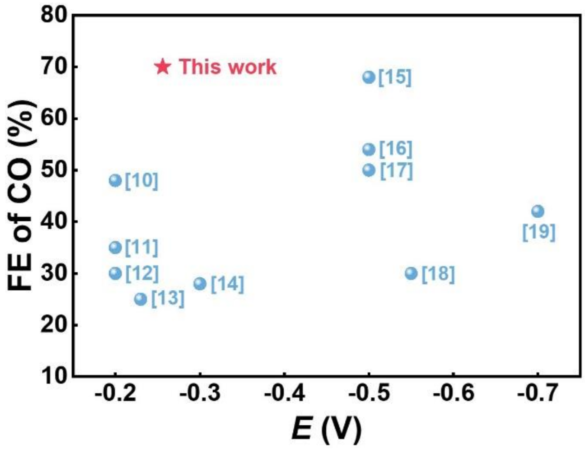

Figure S6. CO_2RR performance of different previously reported catalysts.

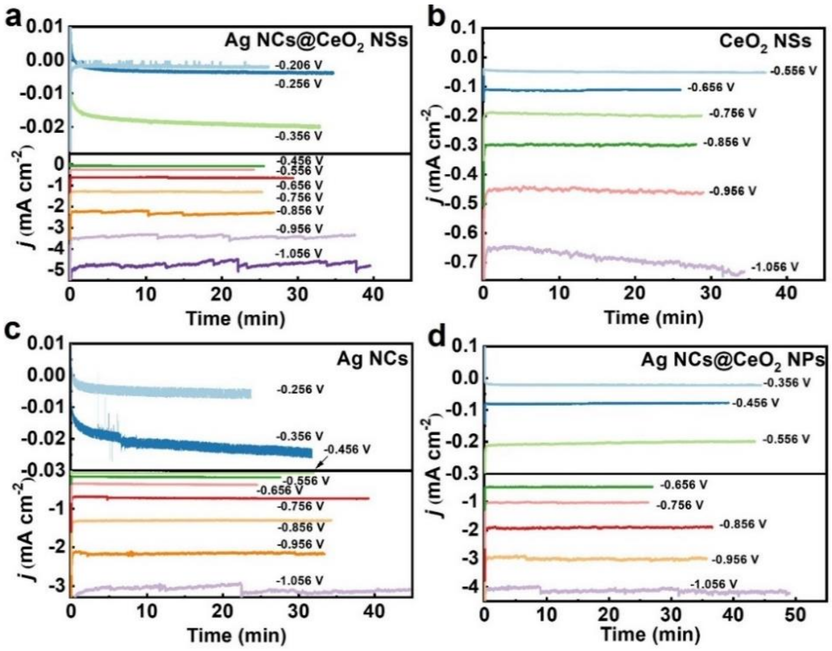

Figure S7. Plots of j as a function of time over (a) Ag NCs@CeO_2 NSs, (b) CeO_2 NSs, (c) Ag NCs and (d) Ag NCs@CeO_2 NPs.

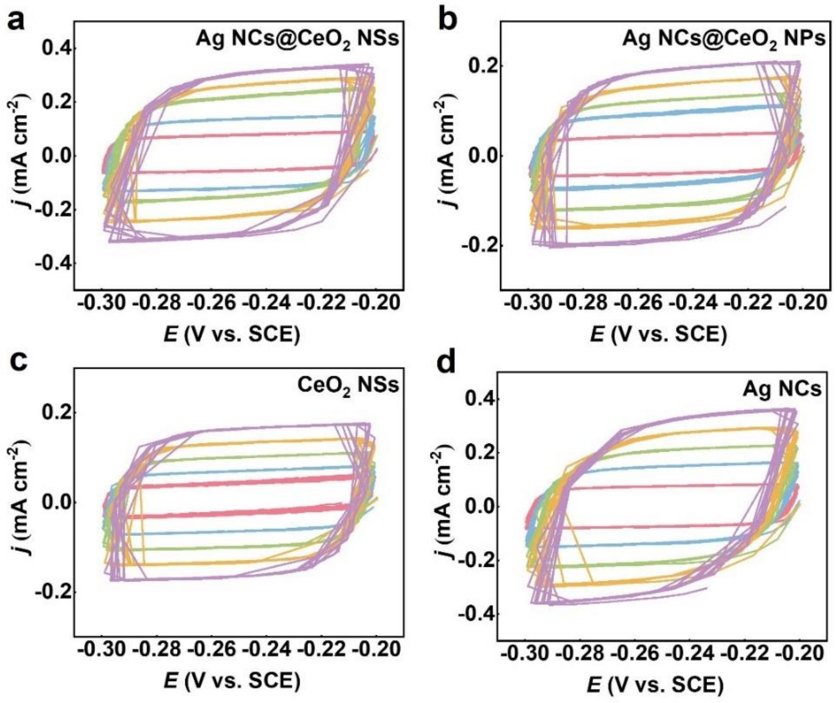

Figure S8. Cyclic voltammetries (CVs) stripping between −0.3 V and −0.2 V (vs. SCE) in CO_2-saturated 0.1 M $KHCO_3$ at scan rates of 20, 40, 60, 80 and 100 mV s^{-1} over (a) Ag NCs@CeO_2 NSs, (b) Ag NCs@CeO_2 NPs, (c) CeO_2 NSs and (d) Ag NCs.

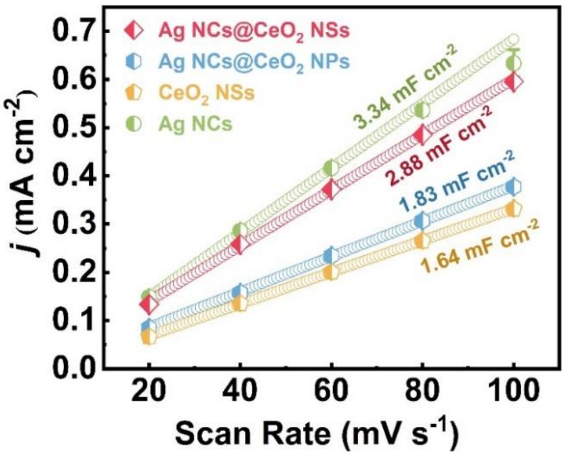

Figure S9. Plots of charging j differences versus scan rates on Ag NCs@CeO$_2$ NSs, Ag NCs@CeO$_2$ NPs, CeO$_2$ NSs and Ag NCs.

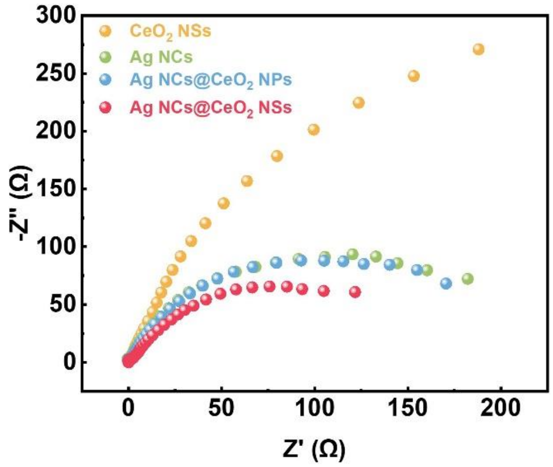

Figure S10. Plots of charging j differences versus scan rates on Ag NCs@CeO$_2$ NSs, Ag NCs@CeO$_2$ NPs, CeO$_2$ NSs and Ag NCs.

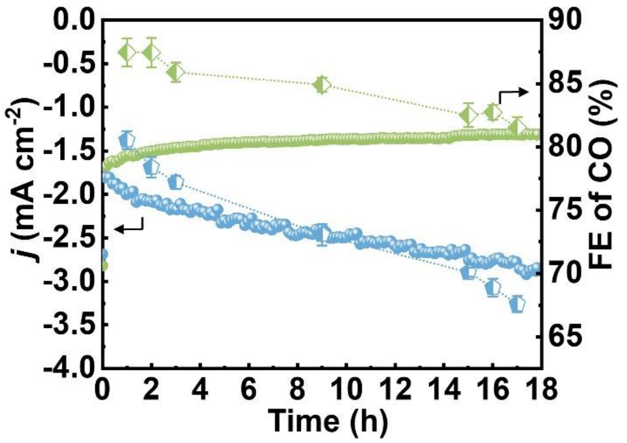

Figure S11. long-term stabilities of Ag NCs and Ag NCs@CeO$_2$ NPs.

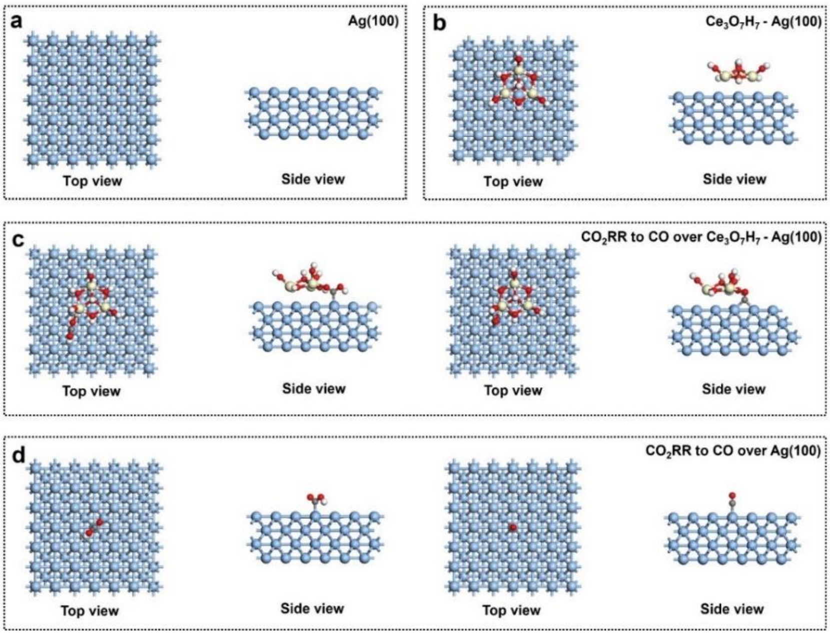

Figure S12. Configurations of optimized (a) Ag(100) and (b) $Ce_3O_7H_7$-Ag(100) models; the adsorption models of *COOH and *CO over (c) $Ce_3O_7H_7$-Ag(100) and (d) Ag(100).

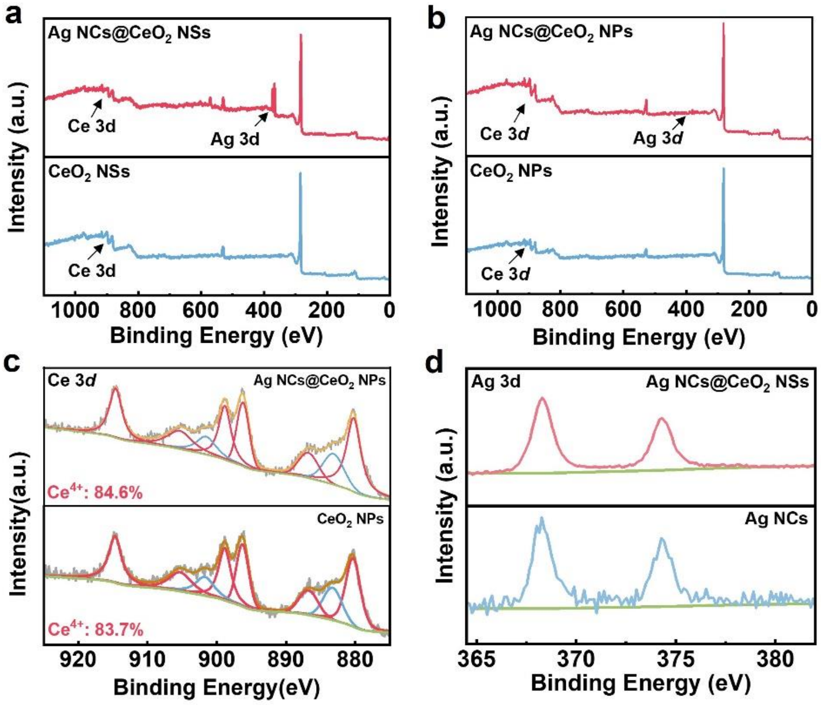

Figure S13. (a, b) Representative XPS of Ag NCs@CeO_2 NSs, CeO_2 NSs, Ag NCs@CeO_2 NPs and CeO_2 NPs; Ce 3*d* of (c) Ag NCs@CeO_2 NPs and CeO_2 NPs and Ag 3*d* of (d) Ag NCs@CeO_2 NSs and Ag NCs.

Supplementary References

[1] J. P. Perdew, K. Burke, M. Ernzerhof, *Phys. Rev. Lett.* **1996**, *77*, 3865.
[2] G. Kresse, J. Furthmüller, *Phys. Rev. B* **1996**, *54*, 11169.
[3] G. Kresse, J. Hafner, *Phys. Rev. B* **1993**, *48*, 13115.
[4] M. Methfessel, A. Paxton, *Phys. Rev. B* **1989**, *40*, 3616.
[5] S. L. Dudarev, G. A. Botton, S. Y. Savrasov, C. Humphreys, A. P. Sutton, *Phys. Rev. B* **1998**, *57*, 1505.
[6] J. A. Rodriguez, P. Liu, J. Hrbek, J. Evans, M. Pérez, *Angew. Chem. Int. Ed.* **2007**, *46*, 1329-1332.
[7] D. Gao, Y. Zhang, Z. Zhou, F. Cai, X. Zhao, W. Huang, Y. Li, J. Zhu, P. Liu, F. Yang, G. Wang, X. Bao, *J. Am. Chem. Soc.* **2017**, *139*, 5652-5655.
[8] A. A. Peterson, F. Abild-Pedersen, F. Studt, J. Rossmeisl, J. K. Nørskov, *Energy Environ. Sci.* **2010**, *3*, 1311-1315.
[9] H. A. Hansen, J. B. Varley, A. A. Peterson, J. K. Nørskov, *J. Phys. Chem. Lett.* **2013**, *4*, 388-392.
[10] M. Liu, M. Liu, X. Wang, S. M. Kozlov, Z. Cao, P. De Luna, H. Li, X. Qiu, K. Liu, J. Hu, *Joule* **2019**, *3*, 1703-1718.
[11] W. Zhu, Y. J. Zhang, H. Zhang, H. Lv, Q. Li, R. Michalsky, A. A. Peterson, S. Sun, *J. Am. Chem. Soc.* **2014**, *136*, 16132-16135.
[12] R. Zhao, Y. Wang, G. Ji, J. Zhong, F. Zhang, M. Chen, S. Tong, P. Wang, Z. Wu, B. Han, Z. Liu, *Adv. Mater.* **2023**, *35*, e2205262.
[13] F. Yang, P. Song, X. Liu, B. Mei, W. Xing, Z. Jiang, L. Gu, W. Xu, *Angew. Chem. Int. Ed.* **2018**, *57*, 12303-12307.
[14] X. Feng, K. Jiang, S. Fan, M. W. Kanan, *J. Am. Chem. Soc.* **2015**, *137*, 4606-4609.
[15] X. Sheng, W. Ge, H. Jiang, C. Li, *Adv. Mater.* **2022**, *34*, e2201295.
[16] D. Gao, Y. Zhang, Z. Zhou, F. Cai, X. Zhao, W. Huang, Y. Li, J. Zhu, P. Liu, F. Yang, G. Wang, X. Bao, *J. Am. Chem. Soc.* **2017**, *139*, 5652-5655.
[17] [S18] M. Ma, B. J. Trzesniewski, J. Xie, W. A. Smith, *Angew. Chem. Int. Ed.* **2016**, *55*, 9748-9752.
[18] Z. Zhang, G. Wen, D. Luo, B. Ren, Y. Zhu, R. Gao, H. Dou, G. Sun, M. Feng, Z. Bai, A. Yu, Z. Chen, *J. Am. Chem. Soc.* **2021**, *143*, 6855-6864.
[19] F. Y. Gao, S. J. Hu, X. L. Zhang, Y. R. Zheng, H. J. Wang, Z. Z. Niu, P. P. Yang, R. C. Bao, T. Ma, Z. Dang, Y. Guan, X. S. Zheng, X. Zheng, J. F. Zhu, M. R. Gao, S. H. Yu, *Angew. Chem. Int. Ed.* **2020**, *59*, 8706-8712.
